# Even Bad Social Norms Promote Positive Interactions

**DOI:** 10.1038/s41598-020-65516-w

**Published:** 2020-05-26

**Authors:** Yoshio Kamijo, Yosuke Kira, Kohei Nitta

**Affiliations:** 1 School of Political Science and Economics, Waseda University, 1-6-1 Nishi-Waseda, Shinjuku-ku, Tokyo, 169-8050 Japan; 2 School of Computer Science and Engineering, The University of Aizu, Aizu-Wakamatsu, Fukushima, 965-8580 Japan; 3 Department of Economics, Chiba University of Commerce, 1-3-1 Konodai, Ichikawa-shi, Chiba, 272-8512 Japan

**Keywords:** Energy and society, Psychology and behaviour

## Abstract

Social norms for cooperation are often supported by positive and negative sanctions. Simultaneously, positive interactions in human relationships via sanctions are promoted by positive social behavior. This study investigates the relationship between social behavior and sanctions based on economic laboratory experiments. Participants with unique IDs make decisions on the contribution to public goods, which is inefficient for society. After participating in the public goods game, they decide whether to use the sanctions. The type of sanctions are varied, such as no sanction, only punishable, only rewardable, and all of these are possible. We found that inefficient social behavior increases under conditions where participants can reward each other and that the level of social activity and rewards are positively correlated. To exclude the possibility of the participants misunderstanding inefficiency, we performed an additional experiment that emphasizes the meaning of inefficiency that the contribution toward public goods reduces profits in society as a whole. We found that even with this emphasis, the high level of contributions is sustained when sanctions are possible. A group-level comparison showed that the group that maintained bad norms used the reward option more. Our results suggest that people maintain bad norms in anticipation of positive interaction.

## Introduction

Some social norms have positive outcomes in society while others have negative outcomes. A typical example of a good social norm is cooperation in situations with a social dilemma such as the preservation of natural resources^1^ and provision of public goods^2^. A cooperative norm deters free-riders and results in better outcomes by indicating a cooperative action as normative behavior. In contrast, some social norms have negative outcomes on welfare. For example, gift giving around the holiday seasons is an event in which people are expected to participate. Although such a custom is considered inefficient by economists^3^, why is this “bad” social norm still prevalent?

Some explanations of bad social norms have been proposed by researchers of economics, sociology, and social psychology both theoretically^4,5^ and experimentally^6–8^, but none have captured the interaction between social norms and social capital. Some researchers view social capital as a mechanism that enhances expected returns of individuals through investment in social relations^9^. If following a bad norm is considered as investment in social capital, returns in the subsequent social interaction are observed. For example, trust in a society is a form of social capital that enables successful social exchange that improves social welfare^10,11^.

Meanwhile, some theorists argue that such norms play a role in developing identity and solidarity in a group^12^. focused on social norms on dress codes and pointed out that members of a social group can “declare one’s group identity to other members and to nonmembers”^13^ proposed that people make gains and losses in accordance and discordance with social identity, and these identity-based payoffs are derived from taking an action that is consistent with one’s social identity. A noteworthy example of this is self-mutilation such as tattooing, body-piercing, and male and female circumcision. None of these have any explicit benefits; instead, they have a cost and they can be painful in some cases. Thus, these can be considered as a sort of inefficient social norm.

Here, we argue that cooperating in inefficient social norms has some positive aspects. They can promote better relationships in some social exchange situations. Some theorists such as^14^ argue that if two different situations are linked, the behavior in one situation affects the behavior in another and vice versa; therefore, decoupling the two situations and analyzing them separately can lead to incorrect conclusions.

To illustrate the interdependent relationship between a bad social norm and enriched human connections, we conducted experiments using an inefficient public goods game (PGG) with punishment and reward options^15^. Similar to a standard PGG, players in the inefficient PGG contribute to a public project and then receive some portion (marginal per capita return, MPCR) of the total contributions of all the players. Contributions to the public project are viewed as pro-social behavior because they increase benefits to all other players at the cost of the contributor. If the MPCR is small, however, they may all become worse off from their contribution to the public project. Therefore, in this game, a social norm that fosters contribution to public goods is a bad social norm that is not worth being protected. Hence, mutual rewards in order to sustain contributions in the PGG are illogical. Nonetheless, if we observe the contribution behavior in this situation, it is reasonable to think that the purpose of the contribution is to promote mutual rewards in the social exchange game (SEG).

We present the results of a series of experiments using an inefficient PGG and SEG. First, we report on the experiment wherein the mutual punishment and the reward opportunity (none of them, only punishments, only rewards, both) after the PGG are manipulated (Study 1). The results revealed that the reward option increases contributions to public goods, and there is a positive correlation between prosocial behavior in the PGG and rewards received in the SEG. Two hypotheses on the motives of the participants can be drawn from these results. Participants might engage in prosocial behavior to increase mutual rewards or they might use rewards to sustain their contribution due to their misunderstanding of inefficiency in the PGG. To eliminate the second possibility, we performed another experiment wherein the inefficiency of the PGG was emphasized via instructions and a test of understanding taken by the participants (Study 2). We find that while this emphasis drastically decreases the contribution when there is no social exchange after the PGG, the contribution is still quite high when there is opportunity for social exchanges after the PGG. A comparison of the groups shows that people in the group who engage in bad social norms are more likely to reward each other and thus, their payoffs are high even though they pay the cost to maintain the inefficient social norm.

## Study 1

We conducted a four-member PGG wherein the participants’ IDs were randomly assigned at the beginning and were fixed throughout the experiment (over 20 repetitions). In each period, they made contributions of 0 to 20 points to a public project where the MPCR was 0.2 and thus, the contribution was socially inefficient (total benefit of one unit of contribution = 0.2*4 = 0.8 < 1 = cost of contribution). Following the PGG, they played the SEG where they were able to punish and/or reward each other after observing their contribution behavior. In the condition with no social exchange (N treatment), there is no sanction option, but there are options for punishment, reward, and both, which are labeled as P, R, and PR treatments, respectively. These options are binary choices: it costs 4 points to punish (reward) a member so that the member loses (gains) 12 points.

Our hypothesis on the reward mechanism states that increased contributions towards inefficient public goods by the reward opportunity is larger than the increase in punishment opportunity under the situation where the participants can identify each of their members by the fixed IDs and thus are able to connect their IDs and their behaviors through the session. That is, our participants encounter a repeated interaction situation and have unique IDs until the end of the session. This is one of our contributions to the literature of inefficient behaviors, which shows that participants increase their contribution towards the inefficient public goods by the punishment opportunity under the situation where the IDs are reshuffled in every period and thus the participants cannot connect with the others’ behaviors beyond the periods^15^. Additionally, the literature covering punishment and reward opportunities in a standard (efficient) public goods game with the fixed IDs revealed that reward opportunity, rather than punishment opportunity, enhances the contribution towards efficient public goods^2,16^. Therefore, our study aims to investigate whether the superiority of the reward opportunity remains in an inefficient public goods situation. This is not a trivial question because inefficient public goods are not socially useful and there is no reasonable explanation to sustain the contribution and no motivation for the reward usage.

## Results

### Contributions at PGG stage

As expected, the amount contributed under the R and PR treatments with the reward option was significantly greater than that for the N and P treatments without the reward option. The group average contributions across 20 periods were used as a data unit, and the effects of the punishment and reward options on the contributions were analyzed using a two-way ANOVA with one factor being the existence of the reward option and the other being the existence of the punishment option. The analysis revealed that the reward option had a significant effect on the contributions (*F*(1, 29) = 4.35, *p* = 0.046). In contrast, the effect of the punishment option (*F*(1, 29) = 1.31, *p* = 0.262) and the interaction of the options (*F*(1, 29) = 0.24, *p* = 0.628) were not significant (Fig. S1 in Supplementary Information for the interaction plot). These results hold even if the data for the final period are not considered. We performed this test because data show some tendency of the end-period effect. In the subsequent analysis, we checked that the statements in the main body hold even when the data of the final period are not considered, if necessary.

**Figure 1:**
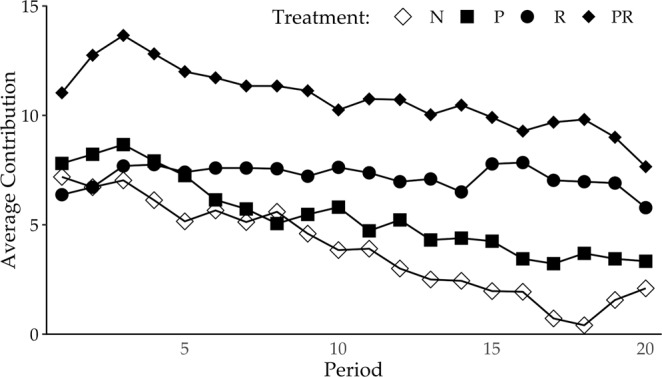
Transition in average contributions across periods per treatment. Note: The amounts for the R and PR treatments are consistently higher than those for the N and P treatments in which participants are unable to give a reward. The trend in the average contribution shows that the amount for the P treatment decreases greatly in later periods, while the amounts for the R and PR treatments are maintained (Table S1 in Supplementary Information). As a result, the difference in the contributions between the R and PR treatments and the N and P treatments becomes more prominent in the later periods.

A comparison of the contribution trends across periods also confirms the positive effect of the reward option on the contribution. The contributions under the R and PR treatments are consistently higher than those under the N and P treatments. In addition, the level of contribution is sustained across periods in the case of the R and PR treatments with the reward option, but it gradually decreases in the condition without the reward option (Fig. 1).

### Payoffs after SEG

In our experimental setting, contributions toward public goods reduce the benefits to society as a whole. Therefore, as indicated by the results from the first stage, the average payoff at the end of the PGG is lower than that when the average contribution is higher. However, the opposite is observed when the results of the SEG are considered (Table 1). That is, although higher contributions are achieved for the R and PR treatments in an inefficient PGG, the final payoffs are higher than those for the other two treatments. A two-way ANOVA revealed a significant effect of the reward option (*F*(1, 29) = 35.81, *p* < 0.001) and the punishment option (*F*(1, 29) = 4.67, *p* = 0.039), and no effect of the interaction term (*F*(1, 29) = 0.03, *p* = 0.855) on the final payoffs.

**Table 1:** Average contribution, sanction use, and payoff in Study 1.

Treatment Name	Contribution	Sanction Use	Payoff
No social exchange	3.88 (3.92)	—	29.22 (0.78)
Punishment	5.40 (6.26)	0.19 (0.20)	25.9 (3.87)
Reward	7.19 (5.51)	1.50 (0.74)	40.56 (6.14)
Punishment-Reward	10.77 (7.68)	1.41 (1.05)	36.56 (7.72)
		P: 0.11 (0.11)	
		R: 1.30 (1.06)	

Note: There are 132 participants and 33 groups. There are 8, 9, 8, and 8 groups under the no social exchange (N), punishment (P), reward (R), and punishment-reward (PR) treatments, respectively. The three columns show the average contribution, sanction use, and payoffs across individuals and periods. The standard deviation at group level is in parentheses.

Higher payoffs in the two treatments with the reward option do not necessarily mean that high contributors in the PGG are rewarded. In our setting, the best way for participants to increase the payoff in the treatment with the reward option is by rewarding each other while ignoring contributions to inefficient public goods. However, the participants did not behave in such a manner, and they seemed to connect the behaviors in the PGG and SEG. Their earnings are positively correlated with the level of contribution in the PR treatment (*r* = 0.80, *p* = 0.017), while there is no correlation in the R treatment (*r* = −0.28, *p* = 0.497), which implies that in the group with a high contribution, the reward option is used sufficiently to compensate for the loss due to the contributions. In other words, they use the reward option in conditional to the contribution level, even though this is an inefficient public good.

### Social exchanges after PGG

To explore how the participants link their behaviors in the PGG and the subsequent SEG, we studied the frequency of the usage of the options and analyzed where the options were used.

The experimental results show that the punishment option is suppressed and the reward option is selected more often (Table 1 and Fig. S2 in Supplementary Information), which proves our hypothesis under an environment with fixed IDs. To promote contributions based on punishments or rewards even in the case of inefficient public goods, the receipt of the punishment or reward should be contingent on the level of contribution of a person. We have already observed that the contribution levels are sustained in treatments with reward options and thus, we predict that the reward option is used for people with high contributions. The results support this prediction. Rewards are used more for a person who has made a high contribution than for one with a low contribution in both the R and PR treatments (Table 2 and Table S2 in Supplementary Information). In addition, the punishment option is directed towards a person with a low contribution, which may seem surprising, given our observation that the level of contribution in the P treatment decreases across the periods. This is due to the low frequency of the usage of punishments compared with rewards. These results are also supported by non-parametric tests (Table S3 in Supplementary Information).

**Table 2:** Who sanctions whom in Study 1 (Punishment-Reward Treatment).

	Punished	Punishing	Rewarded	Rewarding
Constant	0.26^**^	0.21^*^	0.52	0.49
	(0.09)	(0.09)	(0.29)	(0.30)
Others’ Average Contribution	−0.02^**^	−0.01^*^	0.06^***^	0.06^***^
(Amount)	(0.00)	(0.01)	(0.01)	(0.01)
Diff. between Own and Others’ Contributions	−0.02^***^	0.00	0.04^***^	0.01
(Amount)	(0.00)	(0.00)	(0.01)	(0.01)
Period	0.00	0.00	0.01^**^	0.01^*^
	(0.00)	(0.00)	(0.00)	(0.01)
AIC	264.98	563.17	1259.52	1424.76
BIC	296.21	594.40	1290.75	1455.99
Log Likelihood	−125.49	−274.59	−622.76	−705.38
Num. of Observations	640	640	640	640
Num. of Subjects	32	32	32	32
Num. of Groups	8	8	8	8

To see how many punishments/rewards a participant uses/receives per period based on their contribution behaviors in the public goods (1st stage) game, this table shows the results of the analysis of a Linear Mixed Effect model wherein the individual effects and group effects are taken into account by the random effects, and the average of others’ contributions, the difference between the own contribution and the others’ contributions and period effect are fixed effect. The model contains 4 dependent variables including the constant term. The term “Others’ Average Contribution” takes the value of the average contribution by the three other group members (which is excluding the own contribution). The term “Diff. between Own and Others’ Contributions” takes the value of the difference in the contribution between its own contribution and the average contribution by the three other group members. The term “Period” takes the numeric number of the period. The symbols ^***^, ^**^ and ^*^ indicate 0.1%, 1% and 5% significance levels, respectively. “AIC (BIC)” refers to “Akaike (Bayesian)” information criterion.

For the results of the punishment and the reward treatments, please see Table S2 in Supplementary Information. We also calculated the average option usage by participant and determined the difference in the option usage by performing the Mann-Whitney U test (Table S3 in Supplementary Information).

In addition to the conditional usage of punishments and rewards, the response to the usage is also important to sustain the level of contribution in the PGG. Comparisons of the contribution levels before and after receiving punishments or rewards for the first time reveal that for the PR treatment, the reward option had a marginal significant effect on the increase in the contribution, and the punishment option had no significant effect. In contrast, the reward option in the R treatment has no significant effect on the contribution and the punishment option in the P treatment has a marginally significant effect on the contribution (Table 3). Hence, while the significance levels of the reward and punishment options differ among the treatments, we observed that in line with the previous studies of a PGG with sanctions, sanctions have a positive effect on the maintenance or increase in contributions even toward inefficient public goods.

**Table 3:** Effect of sanctions on contributions.

Punishment-Reward Treatment	
Rewarded (only) in the previous period	0.97^+^ (0.10)
Punished (only):	4.00 (0.11)
Rewarded or punished:	1.80^*^ (0.01)
Received neither in the first period:	0.89 (0.67)
Reward Treatment	
Rewarded in the previous period:	0.66 (0.27)
Received no reward in the first period:	1.36 (0.78)
Punishment Treatment	
Punished in the previous period:	2.39^+^ (0.10)
Received no punishment in the first period:	−0.68 (0.82)

Note: The numbers in the right column are the mean of the difference in contribution before and after the punishment/reward was received by individuals. A positive number implies an increase in the contribution after receiving sanctions. For each person, we considered the event when they received a punishment or reward at the first time in order to eliminate the repetitive effect of the punishment or reward and the complicated dynamic aspects as much as possible. The symbols ^*^ and ^+^ indicate 5% and 10% significance levels of the Wilcoxon signed-rank test, respectively.

The results of the usage and response to sanctions are almost the same as those in previous studies on a standard (efficient) PGG. It seems that the efficiency or inefficiency of public goods is irrelevant to the usage of punishments or rewards. This causes some doubt as to whether the participants really understand about the meaning of inefficiency, which was the motivation for our second study.

## Study 2

We investigated why contributions to inefficient public goods are made when the SEG occurs after the PGG. To this end, we manipulated the instructions and emphasized how inefficient one unit of the contribution to public goods is. If the participants still contribute to the public goods after they fully understand the inefficiency and the social loss, we will be convinced that the contribution toward inefficient public goods is an instrumental action that seeks returns in the subsequent stage via rewards. In other words, we explore whether this social bad norm can be considered as an investment in social capital, which will reap benefits in future.

The manipulation of the emphasis has two additional purposes. First, it is a robustness check of Study 1 and the literature^15^. Although the studies in the literature carefully explain the inefficiency of public goods, it is still uncertain whether the participants fully understand the concept of inefficiency as the researchers understand it (i.e., the contribution toward public goods is a socially bad thing because they will be worse off if they all contribute to the public goods). The continued contribution behavior may occur because they do not understand this.

Second, the emphasis on the inefficiency is important to policy makers because it can be considered a mild intervention to abolish a bad social norm. The best scenario is that participants refrain from inefficient behavior with maintaining the reward-based relationship. However, there is a risk that the intervention not only breaks the bad social norms but also reduces the good rewarding behavior. The worst scenario may arise if the participants contribute based on only altruistic motives to society and they are rewarded for the contribution, but the emphasis then destroys such altruistic motives.

To this end, we emphasized the inefficiency of the public goods with and without the reward and punishment options. This manipulation requires the participants to calculate payoffs in the PGG, causing them to inevitably recognize the inefficiency. If the participants pursue their benefits in the SEG and their behavior in the SEG is still contingent on the level of contribution in the PGG, they may contribute in the PGG as well as in the PR treatment of Study 1. We consider four treatments, such as no social exchange with emphasis (NE) and punishment-reward with emphasis (PRE) treatments in which the inefficiency is emphasized, and no social exchange without emphasis (NN) and punishment-reward without emphasis (PRN) treatments in which the inefficiency is not emphasized. In addition, we conducted the pre- and post-experiment surveys to determine the views of the participants on the contributions in the PGG and the options in the SEG, and how these view changed over the course of the experiment.

## Results

### Effect of detailed explanation about inefficiency on contribution behaviors

As shown in Table 4, the reward and punishment options increase the contributions toward public goods, and the emphasis on inefficiency decreases the contributions. In fact, a two-way ANOVA revealed that there is a significant effect of the punishment or reward option (*F*(1, 25) = 25.34, *p* < 0.001), a significant effect of the emphasis (*F*(1, 25) = 6.01, *p* = 0.022), and no effect of the interaction term (*F*(1, 25) = 0.01, *p* = 0.915) on the contributions (Fig. S1 in Supplementary Information for the interaction plot). The change in the contributions per treatment across periods (Fig. S3 in Supplementary Information) shows that high contributions are maintained in the PRN and PRE treatments, but the contributions gradually decline for the NN treatment. In summary, while an emphasis on the inefficiency decreases contributions, bad social norms are practiced and maintained in the treatment with social exchange even with the emphasis.

**Table 4:** Summary of Study 2 (Average of periods).

Treatment	Contribution	Sanctions (R, P)	Payoff
NE	0.44 (0.41)	—	29.91 (0.08)
NN	4.69 (3.60)	—	29.06 (0.72)
PRE	9.30 (7.02)	1.57 (0.64)	36.52 (6.15)
		R: 1.40 (0.72), P: 0.18 (0.14)	
PRN	13.95 (5.21)	1.61 (0.58)	35.51 (6.41)
		R: 1.42 (0.65), P: 0.19 (0.18)	

Note: There are 116 participants and 29 groups. There are 7, 6, 8, and 8 groups of no social exchange with emphasis (NE), no social exchange without emphasis (NN), punishment-reward with emphasis (PRE), and punishment-reward without emphasis (PRN), respectively. The table presents the average of contributions, uses of punishment or reward, and final payoffs across individuals and periods for each treatment. The standard deviation at group level is in parentheses.

### Rewarding and punishing behaviors

Similar to the case in Study 1, high contributors received rewards even though the inefficiency of the contribution was emphasized, while low contributors are punished in the PRE and PRN treatments (Table S4 in Supplementary Information). Similar tendencies to those in Study 1 were observed regarding the effect of the options on the contribution in the subsequent periods. While the reward option in the PRE and PRN treatments had a significant effect on the increase in the contribution, the punishment option had no significant effect in the PRE treatment and a marginal significant effect in the PRN treatment (Table S5 in Supplementary Information).

Overall, the two PR treatments are almost identical in terms of the effect of the punishment or reward option. These results are also similar to those in previous studies of the PGG with sanctions; the sanctions have a positive effect on maintenance of and/or increase in the contribution even toward inefficient public goods.

### Pre- and post-experiment questionnaires

The results of our experiment indicate that the opportunities for social exchange and the emphasis on the inefficiency in the PGG affect social norms for the contribution that the participants perceive.

The questionnaire included the question “how much do you agree with the following opinions?” which had to be answered using a 5-point Likert scale. We chose the following three opinions and determined the average score of them, which was set as the index of social norms for the contribution (the social norm index) perceived by the participants: “contribution to the public project is a duty,” “contribution to the public project is a good thing,” and “there is an expectation that everyone has to contribute to the public project” (Table 5 and Table S6 in Supplementary Information).

**Table 5:** Index of social norms for the contribution of pre- and post-experiment questionnaires.

	NE	NN	PRE	PRN
Before	2.48 (0.81)	3.35 (0.83)	2.85 (1.02)	3.33 (0.87)
After	1.92 (0.85)	2.51 (0.82)	2.73 (1.03)	3.60 (0.96)

Note: The table contains the index of the social norm for the contribution, which are the average scores for the duty-, good- and expectation-related questions in the pre- and post-experiment questionnaires. The standard deviation is in parentheses. We provide detailed information in Table S5 in Supplementary Information.

We found that contribution norms are formed even before the interactions in the first period. In particular, without the emphasis on the inefficiency, the average score of the social norms index in the pre-experiment phase is greater than mid point, which means that participants perceive the contribution norms even before the first period (*p* = 0.049 for NN and *p* = 0.030 for PRN, as per the one-sample Wilcoxon signed-rank test). In addition, the emphasis effectively disrupts the contribution norm irrespective of whether the SEG exists after the PGG (*p* = 0.001 for NE – NN, and *p* = 0.055 for PRE – PRN, as per the Mann-Whitney U test). However, a comparison of the results from the pre- and the post-questionnaires revealed that the social exchange maintains the contribution norm at the initial level (*p* = 0.007 for NE and *p* = 0.001 for NN, *p* = 0.258 for PRE, and *p* = 0.196 for PRN, as per the Wilcoxon signed-rank test). Next, we determined if the participants regarded the contribution norm as a unfavorable one. We compared the scores of the statement “I truthfully did not want to invest” among the experimental treatments, which indicated that while the emphasis on the inefficiency increases the score of unfavorability, the social exchange decreases the score (Table S6 in Supplementary Information). This is consistent with the interpretation that when there is a social exchange after the PGG, participants link the behaviors in the PGG and those in the SEG and thus, they regard the contribution toward the PGG as a kind of investment in social capital that results in returns on the next occasion.

Finally, we examined the reasons behind the social exchange behavior in the SEG and analyzed how these were changed by the emphasis of the inefficiency. In the post-experiment questionnaire, we asked participants using a 5-point Likert scale whether they used the reward and punishment options to ensure fairness, to increase the payoff, out of spite/kindness, or to increase contributions. The results indicate that when there is an emphasis on the inefficiency, participants do not reward in the SEG in order to increase the contribution in the later PGG (Table S7 in Supplementary Information). This also validates our interpretation that they contribute in the PGG in order to obtain the returns from the later SEG.

## Discussion

The two experiments indicated that rewards have a stronger association with an inefficient social norm than do punishments. This is important to the literature on public goods game experiment. First, Studies 1 and 2 have shown that the level of contribution toward inefficient public goods is higher under conditions where participants can reward each other in a fixed ID environment. Alike efficient public goods studies, the reward mechanism hypothesis in inefficient PGG has been examined and verified by our results.

Second, the most important finding in our study is that people cooperate in the PGG to achieve mutual rewards. Previous studies have suggested people reward for the sake of achieving cooperation in the PGG^2,17^. However, participants in Study 2 actively contributed in the PGG and rewarded others mutually even though they understood the inefficiency of the PGG, whereas the emphasis on inefficiency significantly decreased contributions when the reward and punishment options did not exist. This result suggests that people choose to behave altruistically based on prospects for social exchange in another situation.

Inefficient altruistic behavior, which was observed in our experiments, can be considered to include investment in social capital based on the viewpoints of trust and reciprocity in the subsequent social exchange stage^11,18^. Reciprocity might arise when someone behaves kindly and the recipient considers it as kind even if the behavior involves reduced welfare in society. Because contribution toward public goods benefits all members of the group, all of them potentially respond based on reciprocity. Therefore, the linkage of a PGG and dyadic social exchange triggers widespread reciprocity and establishes an effective mechanism for cooperation in later stages.

While we verify that cooperation in a PGG induces rewarding behavior, previous studies have shown that the latter induces the former. We propose that both directions of contribution in a PGG and mutual cooperation in dyadic exchange exist when the provision of public goods is socially efficient. The direction could depend on the proportion of payoffs obtained from the PGG and reward stage. Our experiments assumed that the payoff from mutual rewards is larger than that in the public goods stage (0.8 = the total benefit of one unit of contribution for the PGG vs. 3 for the recipients of rewards). In such cases, the direction from a PGG to a SEG will be stronger than the inverse direction.

The relationship between inefficient public goods and the network of dyadic social exchanges suggests that an attempt to abolish an inefficient social norm requires a deep understanding of the background of social relations in society. The results of our experiments imply that abolishing an inefficient norm is complicated and difficult for policymakers for two reasons. One is that abolition may have negative effects on social exchange networks and result in reduced welfare. The other is that people maintain inefficient norms even if they realize the inefficiency of them, as shown in Study 2. Instead, offering an alternative manner that is less harmful to society could be more effective. Thus, the transformation of a bad norm to less harmful one will be an interesting topic for future research.

Even though our results have a number of potential policy implications, they need to be discussed before making any generalizations. First, the study participants were university students. Although university students and the general public are different in terms of several aspects such as skills, knowledge, and experience^19^, this can be ignored for at least our norm-related task because the Japanese students are almost the same as adults with regard to norm-related dimensions. In addition, aging itself may affect their behaviors and beliefs^20^. have shown that aging is correlated with both prosocial and reciprocal behaviors. Thus, it can be hypothesized that in reality, people have even stronger feelings for sustaining inefficient norms, and future research should test this hypothesis.

Second, we should discuss the cultural difference. In terms of the cooperation and punishment-reward in the PGG^21^, conducted meta-analysis across several countries. Regarding the effect of punishment on cooperative behaviors, they found cultural differences with a variety of impacts by countries and classified Japan as one of the strongest countries. Regarding the effect of reward, there is no clear evidence by countries (Japan has not been found to be unique in this regard). As our main findings pertain to the reward effects to sustain inefficient norms, our findings can be generally expected to be valid.

Third, the four-person group may facilitate mutual rewards based on contribution behavior. The meta-analysis on PGG with punishment or reward^21^ concludes that the group size does not moderate either punishment-cooperation association or reward-cooperation association. However, it appears to be natural that the size of the group wherein each member of the group interacts by using the reward and punishment is smaller than that of the PGG they play. Only a few studies have investigated large group cooperation in PGG through mutual rewards within the smaller group^16,17^, and their results are contrasting (one is positive and another is negative). Thus, future research is required to verify whether our result would hold in a large group setting.

Finally, we should mention that the sample size of our study is relatively small, which causes the small statistical power to detect the small difference. This might be the reason for why the effect of the punishment on the contribution is not statistically significant, being slightly contradicted to some studies on the punishment literature under the fixed ID environment^22,23^. However, our main results about the reward hypothesis are statistically significant even under small sample size because the effect size of the reward conditions is high (Table S8 in Supplementary Information for the effect size of each test and power analysis). In addition, the specific setting such as punishment and reward effectiveness (e.g., the cost ratio of 1:3) may affect our results. To check whether the punishment is effective on the enhancement of the contribution to the inefficient PGG and our results still hold under the different sanction costs are our future researches.

## Methods

### Study 1

Each participant is randomly assigned to a four-member group at the beginning of each session. In each period, they receive an endowment and play two games: an inefficient public goods game (PGG) in the first stage and a social exchange game (SEG) in the second stage. Each participant plays for 20 periods in a session. The composition of the group and the IDs of the members are fixed throughout the experiment. At the end of experiment, we provide a questionnaire to collect the participants’ demographic information and the participants’ impressions of the public goods.

In the PGG, the participants determine how to allocate their endowment towards inefficient public goods where the marginal per capita return is 0.2. One unit of a contribution generates a 0.8 payoff in total; hence, the contribution is inefficient in the sense that such a contribution decreases social welfare. The payoff for each participant is calculated based on the benefit from the public goods and the rest of the endowment.

Following this, the participants play an SEG, and the type of the SEG depends on the experimental conditions. In all the treatments, the results of the contribution decisions in the PGG are made public among the group members. For the control treatment (no social exchange), the participants observe such information and decide to do nothing in this SEG. In other words, they repeatedly play the above PGG, while giving feedback regarding the contribution decisions of the group members.

In order to manipulate the types of social exchange, we consider three types of SEGs. One of the three games is played after the PGG decision in each round. In the P treatment, participants can choose to use or not use a punishment option for each of the other three members. There is no restriction on the number of those whom one member can punish in each round. The punishment option means paying a cost of 4 points for the other member to lose 12 points (this is similar to the setting of^2^). In the R treatment, there is a reward option which means paying a cost of 4 points for the other member to receive 12 points. In the PR treatment, participants have both the punishment and reward options (that is, there are three options of punishment, reward and none) for each of the three other members.

### Study 2

We slightly modify the N and PR treatments of Study 1 by emphasizing and not emphasizing the inefficiency of the public goods, and carry them out in the same manner as in Study 1. As the SEG, we adopt the PR treatment for Study 2, because it has the higher external validity than the P and R treatments and the punishment (reward) usage is similar between the P (R) and the PR treatments. For all treatments, we used pre- and post-experiment questionnaires to determine the participants’ impressions of the public goods. By doing this, we determined the effect of the interaction between participants due to the experimental decisions.

The inefficiency of the public goods was emphasized as follows. Here, inefficiency is not only when the contribution is not enough to increase the profit of others, but also the economic inefficiency that occurs when all the members of the society engage in the contribution, but the benefits to all deteriorate. In order to emphasize the latter, the instruction in the game was modified as follows: “When you invest one unit, the group as a whole loses 0.2 points because you lose 1 point by this investment instead of making a 0.8 profit for the whole group.” Furthermore, in a confirmation test after the instructions, we let the participants understand the meaning of inefficiency via the test. That is, every participant had to calculate the gain when they make the maximum contribution toward public goods (16 points = 20 − 20 + 0.2 × 20 × 4) and when they do not invest (20 points = 20 − 0 + 0.2 × 0 × 4). The four treatments in Study 2 are as follows: the no social exchange with emphasis (NE) and the punishment-reward with emphasis (PRE) treatments in which inefficiency is emphasized, and the no social exchange without emphasis (NN) and the punishment-reward without emphasis (PRN) treatments in which inefficiency is not emphasized. The NN and the PRN treatments in Study 2 are the same as those in Study 1 except for the use of the pre-experiment questionnaire, which is a neutral operation.

### Data collection

The Ethical Review Board of the Kochi University of Technology approved this study. The experiments were carried out in accordance with the approved guidelines. Informed consent was obtained from all participants prior to the experiment.

We conducted the above experiments at the Kochi University of Technology. When implementing a series of experiments, we followed the standard procedures of Experimental Economics. We recruited 132 participants for Study 1 (41 females, 89 males and 2 NAs, 20.08 years old on average, 66 Economics & Management majors and 66 other majors) in December 2017 and January 2018, and 116 participants for Study 2 (36 females and 80 males, 19.34 years old on average, 32 Economics & Management major and 84 other majors) in January, February and April 2018. In recruiting our participants, we used our experimental pool with high anonymity. Each participant separately seated in an individual booth for the entire session. We programmed this experiment with z-Tree^24^ and used neutrally framed statements. For the detailed information, the instruction which we used in our experiment is shown in Supplementary Information. The payment for each participant was varied, depending on their performances, and we paid a fixed show-up fee (900 Japanese yen) to our participants. We paid 1559.3 Japanese yen (*SD* = 146.8) on average including a show-up fee. The conversion rate was 100 points for 100 Japanese yen.

## Supplementary information

Supplementary Information.
