# Supplementary material for: Even Bad Social Norms Promote Positive Interactions
Source: Sci Rep. 2020 May 26;10:8694. doi: 10.1038/s41598-020-65516-w (PMC7251124; doi:10.1038/s41598-020-65516-w)
Supplement: Supplementary file 1 — Supplementary Information. [file 41598_2020_65516_MOESM1_ESM.pdf]

Even Bad Social Norms Promote Positive Interactions

Yoshio Kamijo,[1] Yosuke Kira[2], Kohei Nitta[3*]

[1]School of Political Science and Economics, Waseda University,
1-6-1 Nishi-Waseda, Shinjuku-ku, Tokyo 169-8050, Japan
[2]Deceased
[3]Department of Economics, Chiba University of Commerce,
1-3-1 Konodai, Ichikawa-shi, Chiba 272-8512, Japan
[*]Corresponding author; E-mail: nitta@cuc.ac.jp

Supplementary materials

Figs. S1 to S3

Tables S1 to S8

Experimental Instruction

Figure S1: Interaction Plots

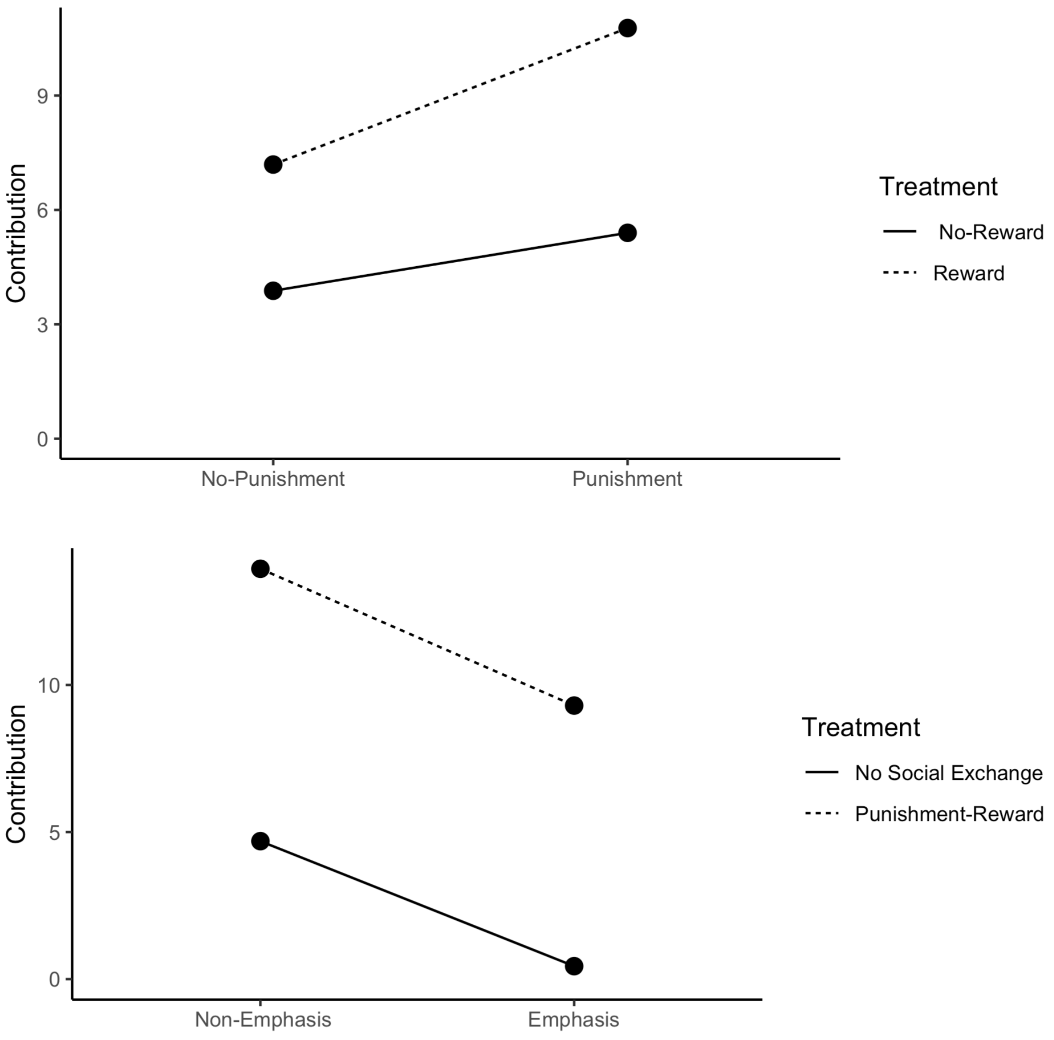

Note: The top and bottom panels show interaction plots of the contribution in Studies 1 and 2, respectively. The amounts contributed in Study 1 are consistently higher when participants are able to give the reward option. The amounts contributed in Study 2 are consistently lower when participants are emphasized how inefficient one contribution to public goods is in the instruction.

Figure S2: Use of Punishment/Reward Options per Treatment

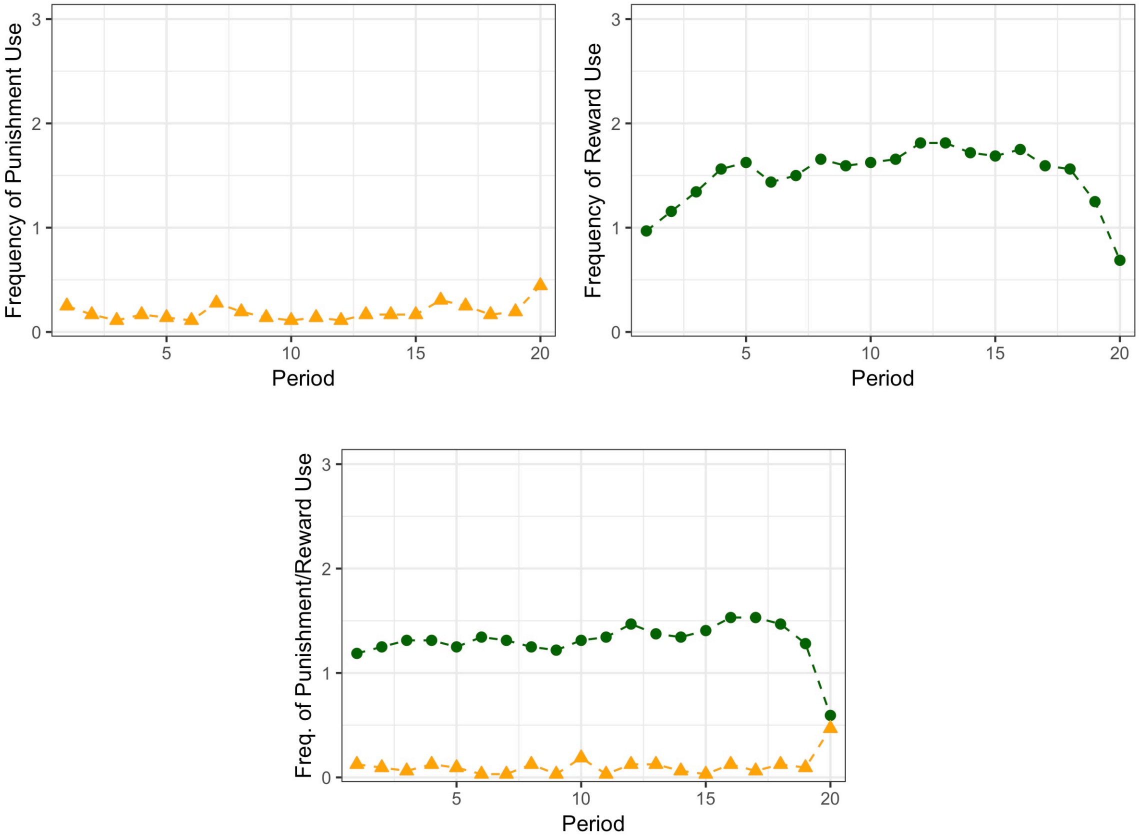

Note: The left and the right panels in the top are the punishment use in the punishment treatment and the reward use in the reward treatment, respectively. The bottom panel is the punishment use and the reward use in the punishment-reward treatment. Each line (yellow line with triangles for punishment, and green line with circles for reward) indicates how many options were used per period by a participant, where the participant was able to use the option toward the members in the same group (max 3 occasions at each option per period).

Figure S3: Transition in average contributions across periods per treatment.

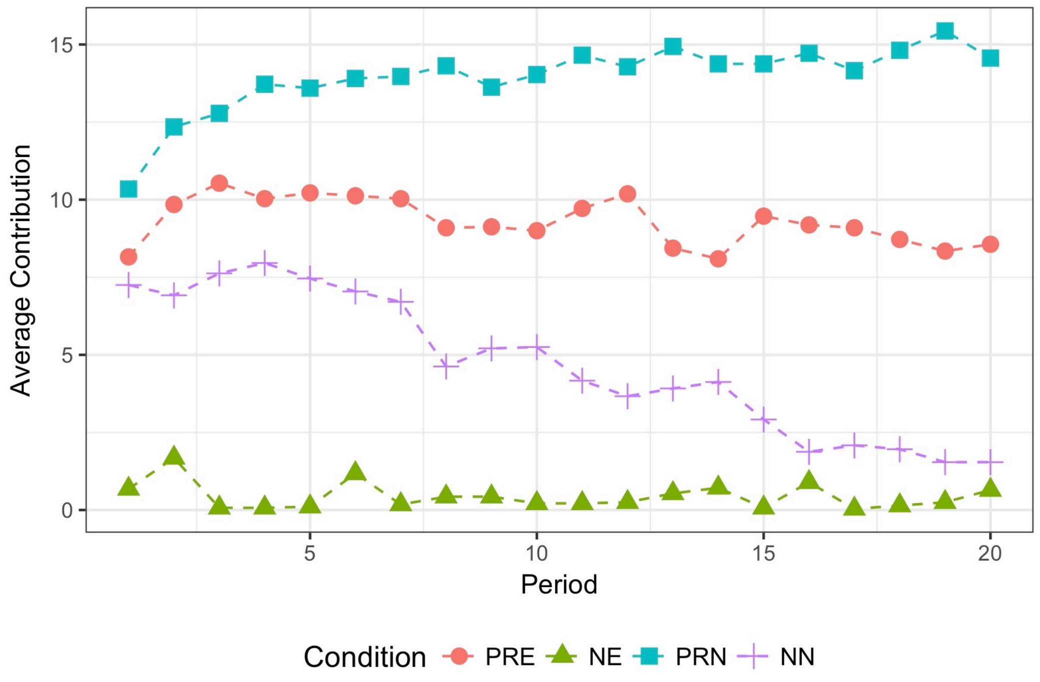

Note: This figure shows the transition in the average contribution per treatment in Study 2. The amounts contributed for the two PR treatments are consistently higher than those for the two N treatments in which participants are unable to give reward and punishment options. The amounts contributed for the two emphasized treatments are lower than those for the two non-emphasized treatments, but there is the significant difference only between the two N treatments. Focusing on the trend in the average contributed amount, the amounts for the two PR treatments are temporally increased in earlier periods. In later periods, the NE treatment decreases gradually (significant at 5% level at the Mann-Whitney U test), while the PRE and NN treatments maintain the same amount of contributions ($n.s.$ by the Mann-Whitney U test). The PRN treatment increases gradually (significant at 10% for 1 vs 20 period and 5 % for 1 vs 19 period at the Mann-Whitney U test).

Table S1: Difference between the contributions of the 1st period and the final period

	1 vs 20	1 vs 19
No Social Exchange Treatment	-5.09^{+} (0.08)	-5.63^{*} (0.02)
Punishment Treatment	-4.47^{*} (0.04)	-4.36^{*} (0.04)
Reward Treatment	-0.59 (1)	0.53 (0.75)
Punishment-Reward Treatment	-3.38 (0.16)	-2.03 (0.40)

Note: Each cell shows the difference in the amount of contributions between 1st and 20th (or 19th) periods across treatments, where we perform the Wilcoxon signed-rank test. The symbols ** , * and $^{+}$ indicate 1%, 5% and 10% significance level, respectively.

Table S2: Who sanctions whom in Study 1 (Linear Mixed Effect Model)

	Punishment		Reward	
	-ed	-ing	-ed	-ing
Constant	0.17	0.17	1.04***	1.04***
	(0.09)	(0.09)	(0.31)	(0.31)
Others' Average Contribution	−0.00	−0.00	0.06***	0.05***
(Amount)	(0.00)	(0.01)	(0.01)	(0.01)
Diff. between Own and Others' Contributions	−0.03***	0.01**	0.05***	0.02**
(Amount)	(0.00)	(0.00)	(0.01)	(0.01)
Period	0.00	0.00	0.01	0.01
	(0.00)	(0.00)	(0.01)	(0.01)
AIC	661.44	820.23	1506.58	1631.40
BIC	693.50	852.29	1537.81	1662.63
Log Likelihood	-323.72	-403.12	-746.29	-808.70

Note: To see how many punishments/rewards a participant uses/receives per period based on their contribution behaviors in the public goods (1st stage) game, this table shows the results of the analysis of a Linear Mixed Effect model wherein the individual effects and group effects are taken into account by the random effects, and the average of others' contributions, the difference between the own contribution and the others' contributions and period effect are fixed effect. While each model in the Punishment treatment has 720 observations, which consist 36 participants and 9 groups, each model in the Reward treatment has 640 observations, which consist 32 participants and 8 groups.

The model contains 4 dependent variables including the constant term. The term "Others' Average Contribution" takes the value of the average contribution by the three other group members (which is excluding the own contribution). The term "Diff. between Own and Others' Contributions" takes the value of the difference in the contribution between its own contribution and the average contribution by the three other group members. The term "Period" takes the numeric number of the period. The symbols ***, ** and * indicate 0.1%, 1% and 5% significance levels, respectively. "AIC (BIC)" refers to "Akaike (Bayesian)" information criterion.

Table S3: Who sanctions whom in Study 1 (Mann-Whitney U test)

	Low Contributions	High Contributions
Punishment-Reward Treatment		
Rewarding	0.97 (1.03)	1.29 (1.20)
Rewarded	0.57 (0.58)	1.36^{*} (1.14)
Punishing	0.07 (0.17)	0.08 (0.19)
Punished	0.23 (0.37)	0.05^{*} (0.18)
Reward Treatment		
Rewarding	1.23 (0.91)	1.63^{+} (0.94)
Rewarded	1.14 (0.65)	1.72^{**} (0.82)
Punishment Treatment		
Punishing	0.09 (0.19)	0.18 (0.38)
Punished	0.34 (0.52)	0.08^{+} (0.14)

Note: Each cell shows how many punishments/rewards a participant uses/receives per period based on their contribution behaviors from 1st to 19th periods. Two types of contributions were established for each period, i.e. high and low contributions based on whether the contribution was above/below the average group contribution. Then, we calculated the average option usage by participant and determined the difference in the option usage. The symbols **, * and $^{+}$ indicate 1%, 5% and 10% significance levels, respectively.

Table S4: Who sanctions whom in Study 2 (PRE & PRN Treatments)

PRE Treatment	Punishment (Left) & Reward (Right)			
	Punished	Punishing	Rewarded	Rewarding
Constant	0.26^{***}	0.24^{**}	0.86^{***}	0.83^{***}
	(0.05)	(0.08)	(0.18)	(0.20)
Others' Average Contribution	-0.01^{***}	-0.01^{*}	0.07^{***}	0.07^{***}
(Amount)	(0.00)	(0.01)	(0.01)	(0.01)
Diff. between Own and Others' Contributions	-0.02^{***}	-0.01^{*}	0.05^{***}	0.03^{**}
(Amount)	(0.00)	(0.00)	(0.01)	(0.01)
Period	0.00^{*}	0.00	−0.01	−0.01
	(0.00)	(0.00)	(0.00)	(0.01)
AIC	490.70	601.73	1228.71	1554.08
BIC	521.93	632.96	1259.94	1585.31
Log Likelihood	-238.35	-293.87	-607.35	-770.04
**PRN Treatment**	**Punishment (Left) & Reward (Right)**			
	Punished	Punishing	Rewarded	Rewarding
Constant	0.56^{***}	0.56^{***}	0.17	0.17
	(0.10)	(0.10)	(0.22)	(0.23)
Others' Average Contribution	-0.03^{***}	-0.03^{***}	0.09^{***}	0.09^{***}
(Amount)	(0.01)	(0.01)	(0.01)	(0.01)
Diff. between Own and Others' Contributions	-0.02^{***}	−0.00	0.03^{***}	0.02^{*}
(Amount)	(0.00)	(0.01)	(0.01)	(0.01)
Period	−0.00	−0.00	−0.00	−0.00
	(0.00)	(0.00)	(0.01)	(0.01)
AIC	641.04	804.61	1461.80	1622.21
BIC	672.27	835.84	1493.03	1653.44
Log Likelihood	-313.52	-395.31	-723.90	-804.11

Note: To see how many punishments/rewards a participant uses/receives per period based on their contribution behaviors in the public goods (1st stage) game, this table shows the results of the analysis of a Linear Mixed Effect model wherein the individual effects and group effects are taken into account by the random effects, and the average of others' contributions, the difference between the own contribution and the others' contributions and period effect are fixed effect. Each model has 640 observations, which consist 32 participants and 8 groups.

The model contains 4 dependent variables including the constant term. The term "Others' Average Contribution" takes the value of the average contribution by the three other group members (which is excluding the own contribution). The term "Diff. between Own and Others' Contributions" takes the value of the difference in the contribution between its own contribution and the average contribution by the three other group members. The term "Period" takes the numeric number of the period. The symbols $^{***}\&^{**}$ and * indicate 0.1%, 1% and 5% significance levels, respectively. "AIC (BIC)" refers to "Akaike (Bayesian)" information criterion.

Table S5: Effect of sanctions on contributions in Study 2

Punish-Reward with Emphasis Treatment (PRE)	
Rewarded (only) in the previous period	: 1.53^{*} (0.04)
Punished (only)	: 1.60 (0.23)
Rewarded or punished	: 1.53^{*} (0.04)
Received neither in first period	: 2.50 (1.00)
Punish-Reward without Emphasis Treatment (PRN)	
Rewarded (only) in the previous period	: 1.25^{*} (0.04)
Punished (only)	: 2.36^{+} (0.10)
Rewarded or punished	: 2.12^{*} (0.01)
Received neither in first period	: 4.00 (0.37)

Note: The numbers in the right column are the mean of the difference in contribution before and after the punishment/reward was received by individuals in Study 2. A positive number implies an increase in the contribution after receiving sanctions. For each person, we considered the event when they received a punishment or reward at the first time in order to eliminate the repetitive effect of the punishment or reward and the complicated dynamic aspects as much as possible. The symbols * and $^{+}$ indicate 5% and 10% significance levels of the Wilcoxon signed-rank test, respectively.

Table S6: Reasons for the contribution of pre- and post-experiment questionnaires

	NE	NN	PRE	PRN
Contribution to the public project is a duty (Duty)				
Before	2.29 (0.90)	2.92 (1.25)	2.48 (1.21)	2.84 (1.30)
After	1.79 (0.99)	2.25 (1.15)	2.50 (1.30)	3.34 (1.38)
Contribution to the public project is a good thing (Good)				
Before	2.68 (1.12)	3.50 (1.25)	3.23 (1.26)	3.66 (1.07)
After	2.43 (1.20)	3.62 (1.21)	2.91 (1.30)	3.59 (1.13)
There is an expectation that everyone has to contribute to the public project (Expectation)				
Before	2.46 (1.10)	3.62 (0.92)	2.84 (1.16)	3.50 (1.11)
After	1.54 (0.92)	1.67 (1.01)	2.78 (1.24)	3.88 (1.21)
I truthfully did not want to invest				
Before	3.68 (1.06)	2.96 (1.30)	3.10 (1.33)	2.25 (0.98)
After	4.18 (0.90)	3.17 (1.31)	3.00 (1.37)	2.44 (1.29)

Note: The table contains the scores of duty-, good- and expectation-related questions in pre-questionnaire per participant, where consist with the social norm score. The scores measure in a 5-point Likart scale, where "5" stands for "strongly agree" and "1" stands for "strongly disagree". The standard deviation at individual level is in parenthesis.

The emphasis effectively disrupts the cooperation norm irrespective of whether the SEG is after the PGG; on duty ($p = 0.051$ for NE-NN, and $p = 0.268$ for PRE-PRN), good ($p = 0.012$ for NE-NN, and $p = 0.171$ for PRE-PRN), and expectation ($p < 0.001$ for NE-NN, and $p = 0.021$ for PRE-PRN) by the Mann-Whitney U test.

Also, the comparisons of the results from the pre- and the post-questionnaires revealed that the social exchange maintains some of the contribution norms at the initial level; on duty ($p = 0.014$ for NE, $p = 0.008$ for NN, $p = 0.923$ for PRE and $p = 0.049$ for PRN), good ($p = 0.535$ for NE, $p = 0.762$ for NN, $p = 0.093$ for PRE and $p = 0.561$ for PRN), and expectation ($p = 0.004$ for NE, $p < 0.001$ for NN, $p = 0.836$ for PRE and $p = 0.340$ for PRN) by the Wilcoxon signed-rank test.

Table S7: Reasons of punishment and reward from post-questionnaire

	PRE	PRN
Punishment for:		
Fairness	3.50 (1.16)	3.72 (1.23)
Increasing Payoff	2.67 (1.18)	2.53 (1.35)
Spite^{+}	2.60 (1.18)	1.89 (1.18)
Increasing Contribution*	2.36 (1.22)	3.56 (1.38)
Rewad for:		
Fairness	3.43 (1.10)	3.55 (1.30)
Increasing Payoff	3.86 (0.92)	3.65 (1.36)
Kindness	3.10 (1.05)	3.20 (1.35)
Increasing Contribution*	2.57 (1.23)	3.45 (1.39)

Note: The table contains the scores by a 5-point Likart scale in post-questionnaire per participant, where "5" stands for "most appropriate" and "1" stands for "most inappropriate". The scores show the results of questions, why the participant punish or reward . The standard deviation at individual level is in parenthesis. The symbols ** , * and $^{+}$ indicate 1%, 5% and 10% significance level, respectively.

By comparing the answers in the PRE and PRN treatments, we found that regarding the reasons behind the reward, the emphasis on the inefficiency decreased the score of increasing the contribution, but there was no significant difference in the contribution for the other three items. In addition, irrespective of the emphasis, the scores of fairness and increasing payoff are significantly greater than the mid point, which indicates that the main reason for rewarding is to increase the payoffs and returns on the contribution ($p < 0.01$ for fairness and increasing payoff, as per the Wilcoxon signed-rank test). In contrast, for the reasons behind the punishment, the emphasis on inefficiency marginally increased the spiteful motivation and decreased the score for increasing the contribution ($p = 0.061$ for spiteful motivation and $p = 0.016$ for increasing payoff, as per the Mann-Whitney U test).

Table S8: Power Analysis

	Effect size	Power
On Contribution in Study 1 (Anova)		
Reward Option	$\eta^2 = 0.125$	0.208
Punishment Option	$\eta^2 = 0.038$	0.550
Interaction term	$\eta^2 = 0.007$	0.078
On Payoff after SEG in Study 1 (Anova)		
Reward Option	$\eta^2 = 0.515$	1
Punishment Option	$\eta^2 = 0.068$	0.582
Interaction term	$\eta^2 = 0.000$	0.054
On Payoff and Contribution in Study 1 (Correlation)		
No Social Exchange Treatment	$r = 1.0000$	1
Punishment Treatment	$r = 0.5998$	0.4283
Reward Treatment	$r = 0.2827$	0.1052
Punishment-Reward Treatment	$r = 0.7990$	0.7287
On Contribution in Study 2 (Anova)		
Punishment/Reward Option	$\eta^2 = 0.450$	0.999
Emphasis	$\eta^2 = 0.107$	0.687
Interaction term	$\eta^2 = 0.000$	0.051
On Pre- and Post-questionnaires in Study 2 (Non-parametric tests)		
Average score of the social norms index (> 3, Signed-rank)		
– No Social Exchange without Emphasis (NN)	$r = 0.2998$	0.4752
– Punishment-Reward without Emphasis Treatment (PRN)	$r = 0.3312$	0.5541
Emphasis disruption on Contribution Norms (Mann-Whitney U)		
– NE-NN Treatments	$r = 0.3775$	0.4013
– PRE-PRN Treatments	$r = 0.2099$	0.1582
Before-After Difference in Social Norm Index (Signed-rank)		
– No Social Exchange with Emphasis (NE)	$r = 0.430$	0.7759
– No Social Exchange without Emphasis (NN)	$r = 0.546$	0.9326
– Punishment-Reward with Emphasis Treatment (PRE)	$r = 0.174$	0.1957
– Punishment-Reward without Emphasis Treatment (PRN)	$r = 0.199$	0.2427

Note: This table shows the results of power analysis for the tests referred to in the main manuscript. All of the scores in the list show the results when the significance level is at 5%.
